# Awake Surgery for a Patient With Glioblastoma and Severe Aphasia: Case Report

**DOI:** 10.1227/neuprac.0000000000000029

**Published:** 2023-02-09

**Authors:** Daisuke Kawauchi, Aiko Matsuoka, Makoto Ohno, Yasuji Miyakita, Masamichi Takahashi, Shunsuke Yanagisawa, Yukie Tamura, Miyu Kikuchi, Takahiro Naka, Tetsufumi Sato, Yoshitaka Narita

**Affiliations:** * Department of Neurosurgery and Neuro-Oncology, National Cancer Center Hospital, Tokyo, Japan;; ‡ Department of Rehabilitation, National Cancer Center Hospital, Tokyo, Japan;; § Department of Anesthesia and Intensive Care, National Cancer Center Hospital, Tokyo, Japan

**Keywords:** Aphasia, Glioblastoma, Awake surgery, Singing, Case report

## Abstract

**BACKGROUND AND IMPORTANCE:**

Patients with severe aphasia rarely become candidates for awake surgery because the intraoperative tasks of awake surgery for patients with aphasia have not been established.

**CLINICAL PRESENTATION:**

A 50-year-old, right-handed woman presented with recurrent glioblastoma invading her left superior temporal gyrus and inferior parietal lobule. She had severe aphasia, as she could barely verbalize her own name. However, we noticed that she could sing nursery rhymes with simple melodies and applied her singing ability as an axis of awake surgery. During awake surgery, she continuously sang simple songs to detect language dysfunction. As a result, 90% of the tumor was resected, preserving her language function and allowing for improvement. She was discharged 9 days after surgery without further neurological deterioration.

**CONCLUSION:**

Awake surgery is usually not indicated in patients with severe aphasia. However, for patients with aphasia who retain the ability to sing, intraoperative singing could be a possible alternative to maximize tumor resection while minimizing neurological dysfunction.

**ABBREVIATIONS:**
ISM: intraoperative stimulation mapping
KPS: Karnofsky Performance Status
SLTA: Standard Language Test of Aphasia
STG: superior temporal gyrus.

Awake surgery is a standard technique for an invasive brain tumor such as glioma near the supratentorial eloquent region. This technique maximizes tumor resection while minimizing neurological deficits such as motor and language dysfunction in patients.

The Japan Awake Surgery Conference established guidelines for awake craniotomy in 2012.^1^ These guidelines recommend awake surgery for patients without apparent aphasia who can fully understand and cooperate with language tasks. Counting numbers, visual naming, auditory comprehension, and free conversation are essential tasks for language mapping. Thus, patients with severe aphasia rarely become candidates for awake surgery.

We present the case of a patient with recurrent glioblastoma (GBM) with severe aphasia who underwent awake surgery. Intraoperative singing could preserve and improve her language function while achieving maximal safe tumor resection.

## CLINICAL PRESENTATION

**FIGURE 1.**
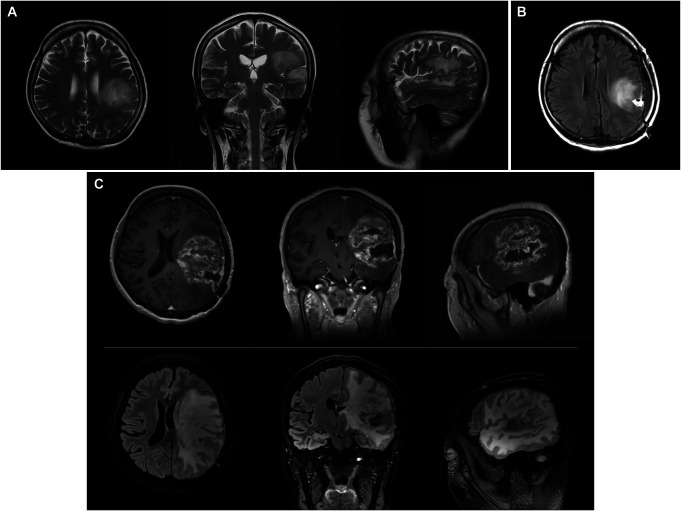
**A**, Axial, coronal, and sagittal fluid-attenuated inversion recovery images taken before the first surgery at a former medical facility. **B**, Axial fluid-attenuated inversion recovery image taken after the first surgery at a former medical facility. **C**, Axial, coronal, and sagittal gadolinium-enhanced T1-weighted (top) and fluid-attenuated inversion recovery (bottom) images taken before the second surgery.

A 50-year-old, right-handed woman presented to our hospital with suspected recurrent GBM or radiation necrosis. She underwent her first awake surgery for a tumor mainly located on the left superior temporal gyrus (STG) and inferior parietal lobule (Figure 1A) at a former medical facility. Intraoperative stimulation mapping (ISM) during the first surgery evoked aphasia in a broad tumor region, including angular and superior marginal gyri. The extent of tumor resection was limited to biopsy (Figure 1B). The pathological diagnosis was a diffuse astrocytic glioma, isocitrate dehydrogenase-wild type, with molecular features of GBM. After the surgery, she received standard radiotherapy (60 Gy in 30 fractions) with concomitant and adjuvant temozolomide. Six months later, an MRI showed that an enlarged gadolinium-enhanced lesion with broad perifocal edema had developed (Figure 1C).

**FIGURE 2.**
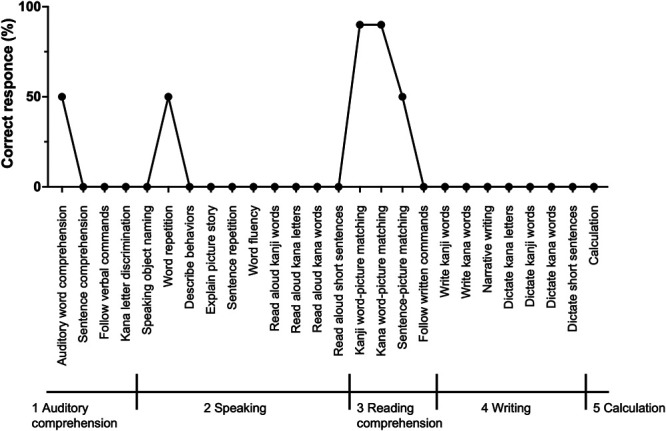
Results of the presurgical SLTA. The total score is 35 points. The maximum score in SLTA is 232 points. SLTA, Standard Language Test of Aphasia.

When this patient first visited our hospital, she presented with severe aphasia, as she could barely speak her name and could not count from 1 to 20. On the Standard Language Test of Aphasia, the most common aphasia evaluation system in Japan, she scored 35 of 232 points (Figure 2). She also had severe right upper limb paralysis and could only open and close her right hand. With 20 mg of prednisolone daily, her preoperative Karnofsky Performance Status (KPS) score was 50. Despite her poor KPS, we planned a second surgery because we expected that debulking the mass lesion and relieving the perifocal brain swelling might improve her neurological dysfunction and prognosis. In addition, the pathological diagnosis was necessary to select the optimal adjuvant therapy. With her severe aphasia, awake surgery along with the general procedure was infeasible. Instead, we noticed that she could sing nursery rhymes with simple melodies such as “Happy Birthday.” We decided to apply her singing ability as a principal intraoperative task for awake surgery.

**FIGURE 3.**
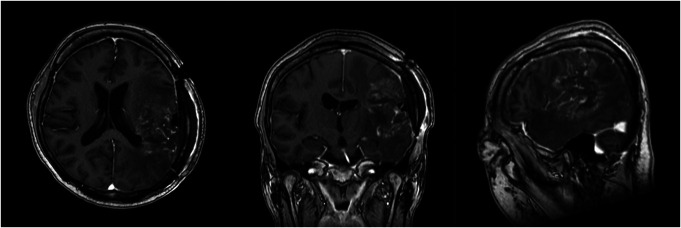
Axial, coronal, and sagittal gadolinium-enhanced T1-weighted images taken 1 day after the second surgery.

She underwent the second awake surgery 9 months after the first surgery. After the craniotomy, the patient was awakened to perform the cortical mapping. ISM was undertaken using bipolar stimulation at 4 mA with an Ojemann stimulator. We asked her to continuously sing nursery rhymes with intermittent right arm movement during ISM. Electrical stimulation on the left STG and inferior parietal lobule did not interrupt her singing but did halt her right arm movement close to the postcentral gyrus. We carefully removed the tumor using low-power SONOPET Ultrasonic Aspirator. As the tumor was resected, her speech function steadily improved, as she could smoothly speak her name and sing songs. When the tumor near the internal capsule was removed, her right extremities became weak, and we finished the awake surgery with 90% tumor resection (Figure 3).

**FIGURE 4.**
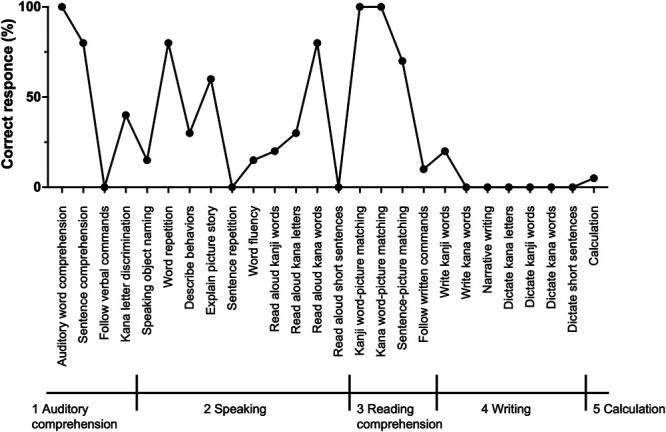
Results of the Standard Language Test of Aphasia taken 7 days after the second surgery. The score is 81 points.

The patient did not experience any severe postsurgical complications. Her aphasia improved, and she could count from 1 to 20. Her postoperative Standard Language Test of Aphasia score improved to 81 points (Figure 4). The right paralysis temporarily worsened but recovered to the preoperative status within 3 days. She was discharged from the hospital 9 days after the surgery with a KPS score of 60. The pathological diagnosis was GBM, isocitrate dehydrogenase-wild type. Postoperative steroid administration tapered off in a month.

**FIGURE 5.**
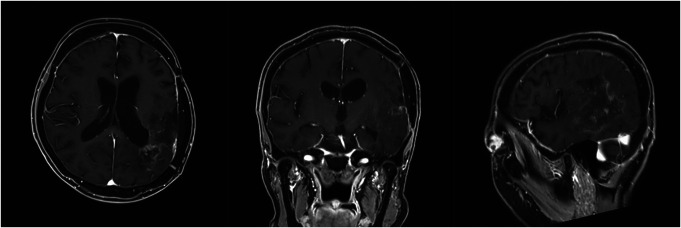
Axial, coronal, and sagittal gadolinium-enhanced T1-weighted images taken 6 months after the second surgery.

She received adjuvant chemotherapy with temozolomide and bevacizumab. Figure 5 shows the MRI image taken 6 months after the surgery. She maintained a KPS score of 70 for 9 months until the third recurrence, which resulted in the deterioration of her neurological status. This study was approved by the internal review board of the National Cancer Center (approval number: 2004-066). Written informed consent was obtained from the patient.

## DISCUSSION

The purpose of awake surgery is to preserve or improve patients' neurological function while maximizing the extent of tumor resection. Therefore, intraoperative tasks should be selected based on patient characteristics and tumor location.^2^ Awake surgery for a patient with severe aphasia is challenging because intraoperative tasks for aphasia patients are not yet established. Our surgical experience expands the possibility of awake surgery, even in patients with severe aphasia, to avoid worsening neurological status.

In this case, the patient could not perform basic tasks for awake surgery, such as counting numbers, visual naming, auditory comprehension, and free conversation. Instead, we chose “singing nursery rhymes” as an axis of language function during the intraoperative task. To the best of our knowledge, this is the first report of awake surgery for an aphasic patient with an atypical task.

The neurofunctional relationship between speaking and singing has long been argued. A recent study demonstrated bilateral activation in the inferior precentral and postcentral gyrus, inferior frontal gyrus, including Brodmann areas 44 and 45, and the middle and posterior portions of the STG and sulcus, during speaking and singing. This result indicates a large integrated network for motor preparation and execution as well as sensory feedback/control for vocal production.^3^ In addition, singing, more so than speaking, showed strong activation in the midportions of the STG, including Heschl gyrus.^3^ Speaking and singing share a large part of the neuronal network.

Because of the difference in neuronal activities, we do not consider that singing would be a compatible task with speaking for patients without aphasia. However, because a large part of a neuronal network is shared, we believe singing could be a possible intraoperative task during awake surgery to preserve or even improve the quality of life of patients with severe aphasia.

### Limitations

In our present case, we demonstrated that intraoperative singing may be an alternative task for severe aphasia patients during awake surgery. However, intraoperative singing is not an established intraoperative procedure. The detailed neuronal mechanism of singing remains unclear. Also, the extent of physical and mental stress for the patient by continuous intraoperative singing is not fully assessed. We must further evaluate intraoperative singing for GBM patients with severe aphasia to establish safe and effective procedures for awake surgery.

## CONCLUSION

Awake surgery is usually not indicated in patients with severe aphasia. However, some patients with aphasia retain the ability to sing. For these patients, intraoperative singing could be an optimal alternative task to prevent further aggravation of aphasia.
